# Co-treatment With BGP-15 Exacerbates 5-Fluorouracil-Induced Gastrointestinal Dysfunction

**DOI:** 10.3389/fnins.2019.00449

**Published:** 2019-05-08

**Authors:** Rachel M. McQuade, Maryam Al Thaalibi, Aaron C. Petersen, Raquel Abalo, Joel C. Bornstein, Emma Rybalka, Kulmira Nurgali

**Affiliations:** ^1^College of Health & Biomedicine, Victoria University, Melbourne, VIC, Australia; ^2^Institute for Health & Sport, Victoria University, Melbourne, VIC, Australia; ^3^Australian Institute for Musculoskeletal Science, Melbourne, VIC, Australia; ^4^Área de Farmacología y Nutrición y Unidad Asociada al Instituto de Química Médica del Consejo Superior de Investigaciones Científicas, Universidad Rey Juan Carlos, Alcorcón, Spain; ^5^Department of Physiology, The University of Melbourne, Melbourne, VIC, Australia; ^6^Department of Medicine Western Health, The University of Melbourne, Melbourne, VIC, Australia; ^7^Head of Enteric Neuropathy Lab, Western Centre for Health Research and Education, Sunshine Hospital, Melbourne, VIC, Australia

**Keywords:** chemotherapy, enteric neurons, 5-fluorouracil, BGP-15, neuroprotection, cytoprotection

## Abstract

Gastrointestinal (GI) side-effects of chemotherapy present a constant impediment to efficient and tolerable treatment of cancer. GI symptoms often lead to dose reduction, delays and cessation of treatment. Chemotherapy-induced nausea, bloating, vomiting, constipation, and/or diarrhea can persist up to 10 years post-treatment. We have previously reported that long-term 5-fluorouracil (5-FU) administration results in enteric neuronal loss, acute inflammation and intestinal dysfunction. In this study, we investigated whether the cytoprotectant, BGP-15, has a neuroprotective effect during 5-FU treatment. Balb/c mice received tri-weekly intraperitoneal 5-FU (23 mg/kg/d) administration with and without BGP-15 (15 mg/kg/d) for up to 14 days. GI transit was analyzed via *in vivo* serial X-ray imaging prior to and following 3, 7, and 14 days of treatment. On day 14, colons were collected for assessment of *ex vivo* colonic motility, neuronal mitochondrial superoxide, and cytochrome *c* levels as well as immunohistochemical analysis of myenteric neurons. BGP-15 did not inhibit 5-FU-induced neuronal loss, but significantly increased the number and proportion of choline acetyltransferase (ChAT)-immunoreactive (IR) and neuronal nitric oxide synthase (nNOS)-IR neurons in the myenteric plexus. BGP-15 co-administration significantly increased mitochondrial superoxide production, mitochondrial depolarization and cytochrome *c* release in myenteric plexus and exacerbated 5-FU-induced colonic inflammation. BGP-15 exacerbated 5-FU-induced colonic dysmotility by reducing the number and proportion of colonic migrating motor complexes and increasing the number and proportion of fragmented contractions and increased fecal water content indicative of diarrhea. Taken together, BGP-15 co-treatment aggravates 5-FU-induced GI side-effects, in contrast with our previous findings that BGP-15 alleviates GI side-effects of oxaliplatin.

## Introduction

Colorectal cancer (CRC) is a significant contributor to cancer related mortality, resulting in more than 1 million deaths annually (World Health Organization, 2011; Ferlay et al., 2015). Due to its asymptomatic nature, approximately 60% of CRC sufferers are diagnosed at or beyond stage III and a positive prognosis is heavily dependent upon chemotherapeutic treatment (Siegel et al., 2014). For several decades after its discovery, the antimetabolite 5-fluorouracil (5-FU) was the only available chemotherapeutic agent shown to improve 12-month survival rates in CRC patients. To date, 5-FU remains the backbone of CRC therapy (Goldberg, 2005), and when administered in combination with oxaliplatin and leucovorin (FOLFOX), or irinotecan and leucovorin (FOLFIRI), comprises one of the most successful treatment regimens for CRC (Liang et al., 2010; Ziauddin et al., 2010).

Over the past 30 years important modulation strategies have been developed to increase anti-cancer activity and overcome clinical resistance associated with 5-FU therapy. However, an array of side-effects including stomatitis, esophago-pharyngitis, alopecia, hematopoietic depression, fever, cardiotoxicity, neurotoxicity, diarrhea, nausea, and vomiting persist as major dose-limiting toxicities (Curreri et al., 1958; Diasio and Harris, 1989; Kennedy, 1999). Gastrointestinal side-effects in particular are prevalent in patients receiving dual administration of 5-FU and leucovorin with 15–20% suffering severe diarrhea as a result of lower gastrointestinal tract toxicity (Buroker et al., 1994; Fata et al., 1999).

It has been suggested that 5-FU-induced gastrointestinal dysfunction results from inflammation, epithelial degradation, and intestinal ulceration triggering intestinal mucositis (Duncan and Grant, 2003). However, a recent study undertaken by our group, showed that acute intestinal inflammation observed at day 3 is resolved by day 7 but gastrointestinal dysfunction persists throughout the course of treatment (McQuade et al., 2016b). Our findings highlight inflammation-induced myenteric neuronal loss as a potential contributing factor to persistent 5-FU-induced gastrointestinal dysfunction (McQuade et al., 2016b). It has been shown that inflammatory cells liberate ROS at the site of inflammation leading to oxidative stress (Biswas, 2016), and initiate intracellular signaling cascades that enhance pro-inflammatory gene expression (Anderson et al., 1994; Flohé et al., 1997). 5-FU-induced apoptosis is correlated with intracellular oxidative stress in many cell types (Hosokawa et al., 2007; Lamberti et al., 2012) and we have shown that oxaliplatin-induced oxidative stress is correlated with neuronal loss (McQuade et al., 2016a).

The cytoprotective agent BGP-15 (C_14_H_22_N_4_O_2_ 2HCl) has demonstrated neuroprotective efficacy in animal models of chemotherapy-induced peripheral neurotoxicity (Bardos et al., 2003). Our recent work has shown that BGP-15 co-treatment alleviates neuropathy and gastrointestinal dysfunction associated with oxaliplatin administration (McQuade et al., 2018). Given that 5-FU-induced inflammation was resolved by day 7, but all other changes persisted long-term, we investigated the effects of *in vivo* long-term BGP-15 co-treatment with 5-FU. In this study we examined (i) *in vivo* gastrointestinal transit and emptying, (ii) fecal water content, (iii) colonic inflammation, (iv) *ex vivo* colonic motility functions, (v) neuronal mitochondrial superoxide and cytochrome *c* levels in myenteric ganglia, and (vi) the total number of myenteric neurons, and subpopulations of inhibitory and excitatory neurons. We aimed to determine the effect of 5-FU treatment on these measures and the capacity for BGP-5 to protect against gut dysfunction.

## Materials and Methods

### Ethics Statement

All procedures were approved by the Victoria University Animal Experimentation Ethics Committee and performed in accordance with the guidelines of the National Health and Medical Research Council (NHMRC) Australian Code of Practice for the Care and Use of Animals for Scientific Purposes.

### Animals

Male Balb/c mice aged 6–8 weeks (18–25 g) supplied by the Animal Resources Centre (Perth, Australia) were used for the experiments. Mice had free access to food and water and were kept under a 12-h light/dark cycle in a well-ventilated room at an approximate temperature of 22°C. Mice were acclimatized for at least 5 days and up to 7 days prior to the commencement of *in vivo* intraperitoneal injections of chemotherapy. A total of 40 mice were used for this study.

### Intraperitoneal Injections

Mice received intraperitoneal injections of 5-FU (23 mg/kg) (Sigma-Aldrich, Australia), with or without BGP-15 (15 mg/kg) (N-Gene R&D Inc., United States) three times a week via a 26-gauge needle as previously described (McQuade et al., 2016a, 2018; McQuade, 2017). 5-FU and BGP-15 were dissolved in 100% DMSO (Sigma-Aldrich, Australia) to make 1 M L^-1^ stock solution refrigerated at -20°C. The stock was then defrosted and diluted with sterile water to make 0.1 M L^-1^ (10% DMSO) solutions for intraperitoneal injections. The dose of 5-FU was calculated to be equivalent to standard human dose per body surface area (Reagan-Shaw et al., 2008). Sham-treated mice received 10% DMSO in sterile water via intraperitoneal injection three times a week via a 26-gauge needle. A separate cohort of mice received BGP-15 (15 mg/kg) alone via intraperitoneal injection three times a week via a 26-gauge needle (McQuade, 2017). The injected volumes were calculated according to body weight; the maximum volume did not exceed 200 μL per injection. At the conclusion of treatment, mice were euthanized via cervical dislocation at either 3, 7, or 14 days after the first injection, and the colons were collected for *in vitro* experiments.

### Assessment of Mitochondrial Superoxide Production

MitoSOX^TM^ Red M36008 (Invitrogen, Australia), was used to visualize mitochondrially derived superoxide in wholemount preparations of myenteric ganglia of the colon. Freshly excised distal colon preparations were dissected to expose myenteric plexus. Preparations were incubated in oxygenated physiological saline with MitoSOX^TM^ Red M36008 (5 μM) at a constant temperature of 37°C for 40 min with gentle agitation. Tissues were washed (2 × 30 min) with oxygenated physiological saline (composition in mM: NaCl 118, KCl 4.6, CaCl_2_ 3.5, MgSO_4_ 1.2, NaH_2_PO_4_ 1, NaHCO_3_ 25, D-Glucose 11; bubbled with 95% O_2_ and 5% CO_2_) and fixed in 4% paraformaldehyde (1.3 M) overnight at 4°C. The following day, tissues were washed (2 × 30 min) with physiological saline and mounted on glass slides with DAKO fluorescent mounting medium for imaging under a Nikon Eclipse Ti laser scanning confocal microscope (Nikon, Japan). All images were captured at identical acquisition exposure-time conditions, calibrated to standardized minimum baseline fluorescence, converted to binary and changes in fluorescence from baseline were measured in arbitrary units (arb. units) using Image J software (NIH, MD, United States). The corrected total fluorescence was calculated as previously described (Burgess et al., 2010) in 32 5 μm × 5 μm boxes within myenteric ganglia from each preparation to exclude fluorescence outside the ganglia (McQuade, 2017).

### Mitochondrial Membrane Potential Assay

Mitochondrial membrane potential changes in cells can be detected with the use of the cationic, lipophilic dye JC-10. In normal cells, JC-10 concentrates in the mitochondrial matrix where it forms red fluorescent aggregates (JC aggregates). In contrast, apoptotic cells stain in green fluorescent color (JC monomeric form) due to the JC-10-labeled release of cytochrome *c* diffusing out of the mitochondria as a result of mitochondrial depolarization and increased permeability. A JC-10 fluorescent mitochondrial membrane potential microplate assay kit (Abcam, MA, United States) was used to detect mitochondrial membrane potential changes and the release of cytochrome *c* from damaged mitochondria in the myenteric ganglia of the colon. Freshly excised distal colon preparations from DMSO and 5-FU-treated mice were bathed in oxygenated physiological saline and dissected to expose the myenteric ganglia. Immediately following dissection, preparations were incubated for 20 min with 500 μL of JC-10 dye solution (buffer A) and gently agitated at a constant temperature of 37°C. After 20 min, 500 μL of buffer B solution was added to tissue preparations and allowed to incubate for another 20 min with constant agitation at a constant temperature of 37°C. Immediately following the final incubation, tissues were mounted on glass slides with DAKO fluorescent mounting medium for imaging under a Nikon Eclipse Ti laser scanning confocal microscope (Nikon, Japan) (McQuade, 2017).

### Immunohistochemistry in Wholemount Preparations

Colon segments (2–3 cm) were placed in oxygenated phosphate-buffered saline (PBS) (pH 7.2) containing nicardipine (3 μM) (Sigma-Aldrich, Australia) for 20 min to inhibit smooth muscle contractions. They were then cut open along the mesenteric border, cleared of their contents, maximally stretched and dissected mucosa up to expose the myenteric plexus attached to the longitudinal muscle layer. Tissues were fixed with Zamboni’s fixative (2% formaldehyde and 0.2% picric acid) overnight at 4°C. Preparations were cleared of fixative by 3 × 10 min washes with DMSO (Sigma-Aldrich, Australia) followed with 3 × 10 min washes with PBS. Fixed tissues were stored at 4°C in PBS for a maximum of 5 days (McQuade, 2017).

Wholemount preparations were incubated with 10% normal donkey serum (Chemicon, United States) for 1 h at room temperature, then washed (2 × 5 min) with PBS and incubated with primary antibodies against β Tubulin III (βTubIII) (chicken, 1:1000, Abcam, MA, United States), nNOS (goat, 1:500, Abcam, MA, United States), and choline acetyl transferase (ChAT) (goat, 1:200, Abcam, MA, United States) overnight at 4°C. Tissues were then washed in PBS (3 × 10 min) before incubation with species-specific secondary antibodies labeled with different fluorophores: donkey anti-chicken Alexa 594 (1:200, Jackson ImmunoResearch Laboratories, PA, United States) and donkey anti-goat Alexa 488 (1:200, Jackson ImmunoResearch Laboratories, PA, United States) for 2 h at room temperature. Wholemounts were given 3 × 10 min final washes in PBS and mounted on glass slides using fluorescence mounting medium (DAKO, Australia) (McQuade, 2017).

### Immunohistochemistry in Cross Sections

Colon sections (1–2 cm) were placed in oxygenated PBS containing nicardipine (3 μM) (Sigma-Aldrich, Australia) for 20 min to inhibit smooth muscle contractions. Samples were cut open along the mesenteric border, cleared of their contents, and pinned mucosa up without stretching. Tissues were fixed with Zamboni’s fixative overnight at 4°C. Preparations were cleared of fixative by washing 3 × 10 min with DMSO (Sigma-Aldrich, Australia) followed by 3 × 10 min washes with PBS. After washing, tissues were embedded in 100% OCT and frozen using liquid nitrogen (LN_2_) and isopentane (2-methyl butane) and stored in -80°C freezer. Tissues were cut at 20 μm section thickness using a Leica CM1950 cryostat (Leica Biosystems, Germany), adhered to slides and allowed to rest for 30 min at room temperature before processing (McQuade, 2017).

Cross section preparations were incubated with 10% normal donkey serum (Chemicon, United States) for 1 h at room temperature. Tissues were then washed (2 × 5 min) with PBS and incubated with primary antibody against CD45 (rat, 1:500, BioLegend, Australia) overnight at 4°C. Sections were then washed in PBS (3 × 10 min) before incubation with secondary antibody labeled with fluorophore donkey anti-rat Alexa 488 (1:200, Jackson ImmunoResearch Laboratories, PA, United States) for 2 h at room temperature. The sections were given 3 × 10 min final washes in PBS and then cover-slipped using fluorescence mounting medium (DAKO, Australia). Sections were viewed under a Nikon Eclipse Ti laser scanning confocal microscope (Nikon, Japan), whereby eight randomly chosen images from each preparation were captured with a 20× objective and processed using NIS Elements software (Nikon, Japan). The number of CD45+ IR cells was quantified within a 2 mm^2^ area in every colonic section (McQuade, 2017).

### Imaging

Three dimensional (z-series) images of wholemount preparations were taken using a Nikon Eclipse Ti laser scanning microscope (Nikon, Japan), eight randomly chosen images from each preparation were captured with a 20× objective and processed using NIS Elements software (Nikon, Japan). The number of βTubIII, nNOS, and ChAT IR neurons was quantified in the myenteric ganglia within a 2 mm^2^ area of each preparation. Z-series images were taken at a step size of 1.75 μm (1600 × 1200 pixels). All slides were coded and analyzed blind (McQuade et al., 2016a, 2018; McQuade, 2017).

### Gastrointestinal Transit

Gastrointestinal transit was studied by X-ray before the first treatment (day 0) and after 3, 7, and 14 days of 5-FU ± BGP-15 treatment. The contrast agent, 0.4 mL of suspended barium sulfate (X-OPAQUE-HD, 2.5 g/mL), was administered via oral gavage. Prior to performing X-ray imaging, animals were trained/conditioned for oral gavage using a non-irritating substance 0.9% w/v saline (volume 0.1–0.4 ml); this was repeated at least three times with each animal with at least 24 h between each training (McQuade, 2017). Radiographs of the gastrointestinal tract were performed using a HiRay Plus Porta610HF X-ray apparatus (JOC Corp., Kanagawa, Japan; 50 kV, 0.3 mAs, exposure time 60 ms). Mice were immobilized in the prone position by placing them inside a transparent plastic restraint tube with a partly open front side for breathing, which comfortably restrains animal movement essential for maximum of 1–2 min for successful X-ray imaging. The training/conditioning with restraint was achieved by placing the restrainer into the mouse cages for at least 24 h prior to the X-ray procedure. X-rays were captured using Fujifilm cassettes (24 × 30 cm) immediately after administration of barium sulfate (T0) then every 5 min for the first hour, every 10 min for the second hour, and then every 20 min through to 360 min (T360). Animals were closely monitored during and after all procedures. Images were developed via a Fujifilm FCR Capsula XLII and analyzed using eFilm 4.0.2 software. Speed of gastrointestinal transit was calculated as time in minutes taken to reach each region of the gastrointestinal tract (stomach, small intestines, caecum, and large intestines). Organ emptying was calculated as the time taken for complete barium emptying from specific gastrointestinal regions (stomach, small intestines) (Cabezos et al., 2008, 2010; Girón et al., 2015).

### Colonic Motility Experiments

The entire colon was removed from mice and set up in organ-bath chambers to record motor patterns *in vitro* (Wafai et al., 2013). Briefly, the colon was placed into warmed (35°C), oxygenated physiological saline until the fecal pellets were expelled. The empty colon was cannulated at both ends and arranged horizontally in organ-bath chambers. The proximal end of the colon was connected to a reservoir containing oxygenated physiological saline to maintain intraluminal pressure. The distal end was attached to an outflow tube that provided a maximum of 2 cm H_2_O back-pressure. Organ baths were continuously superfused with oxygenated physiological saline solution and preparations were left to equilibrate for 30 min. Contractile activity of each segment was recorded with a Logitech Quickcam Pro camera positioned 7–8 cm above the preparation. Videos (2 × 15 min) of each test condition were captured and saved in *avi* format using VirtualDub software (version 1.9.11) (McQuade et al., 2016a, 2018; McQuade, 2017).

CMMCs were defined as propagating contractions directed from the proximal to the distal end of the colon which traveled more than 50% of the colon length (Roberts et al., 2007, 2008; McQuade et al., 2016b; McQuade, 2017). Contractions that propagated less than 50% colon length were considered to be SCs. Another form of incomplete contraction was identified as FCs occurring simultaneously at different parts of the colon rather than propagating over the length of the colon (McQuade et al., 2016b). Recordings were used to construct spatiotemporal maps using in-house edge detection software (Gwynne et al., 2004). Spatiotemporal maps plot the diameter of the colon at all points during the recording allowing contractile motor patterns to be analyzed with Matlab software (version 12).

### Fecal Water Content

Mice were single housed in cages without bedding or food with free access to water for 20 min on days 3, 7, and 14 of treatment, between 9 and 10 am. Feces were collected for analysis. Wet weight of fecal pellets was measured immediately upon pellet expulsion. Pellets were then dehydrated for 72 h at room temperature prior to measurement of the dry weight. Water content was calculated as the difference between the wet weight and dry weight (McQuade, 2017).

### Statistical Analysis

Data were assessed using two-way ANOVA, Welch’s two-tailed *t*-test and Student’s two-tailed *t*-test. Analyses were performed using Graph Pad Prism (Graph Pad Software Inc., CA, United States). Data are presented as mean ± standard error of the mean (SEM). Value differences were considered statistically significant at *P* < 0.05.

## Results

### Co-administration of BGP-15 With 5-FU Delays Gastrointestinal Transit

To determine the effects of 5-FU treatment with and without BGP-15 on gastrointestinal transit, a series of radiographic images were used to track barium sulfate through the gastrointestinal tract at day 3, 7, and 14 following treatment with DMSO, BGP-15, 5-FU, or 5-FU+BGP-15. The speed of barium movement was calculated by tracing barium entry from one part of the gastrointestinal tract to the next.

After 3 days of 5-FU administration, movement of barium to the caecum and colon, gastric emptying, intestinal emptying and pellet formation occurred significantly faster when compared to DMSO-treated mice (Supplementary Figures S1A–E and Supplementary Table S1). Treatment with BGP-15 alone significantly sped up movement of barium to the caecum; but had no effect on the speed of barium movement within the colon, or on gastric emptying, intestinal emptying or pellet formation when compared to DMSO-treated mice (Supplementary Figures S1A–E and Supplementary Table S1). When compared to 5-FU-treated mice, BGP-15 treatment did not alter movement of barium to the caecum, but significantly delayed movement of barium in the colon and delayed gastric emptying, intestinal emptying and pellet formation (Supplementary Figures S1A–E and Supplementary Table S1). When given together with 5-FU, BGP-15 treatment significantly delayed movement of barium to both the caecum and colon as well as delaying gastric emptying and pellet formation when compared to 5-FU-treated mice (Supplementary Figures S1A–E and Supplementary Table S1). When compared to the DMSO-treated mice, there was no significant differences in gastric emptying, intestinal emptying or pellet formation in 5-FU+BGP-treated mice (Supplementary Figures S1A–E and Supplementary Table S1).

After 7 days of 5-FU administration, movements of barium within the colon, gastric emptying, intestinal emptying and pellet formation were all significantly delayed when compared to DMSO-treated mice (Supplementary Figures S2A–E and Supplementary Table S2). When administered with 5-FU, BGP-15 treatment had no effect on the movement of barium in the caecum and colon, intestinal emptying or pellet formation but did significantly delay gastric emptying when compared to DMSO-treated mice. When compared to 5-FU-treated mice, 5-FU+BGP-15-treated mice had significantly faster barium movement through the colon, but not the caecum, and pellet formation occurred significantly faster, albeit gastric, and intestinal emptying were not different (Supplementary Figures S2A–E and Supplementary Table S2). Treatment with BGP-15 alone significantly sped up the movement of barium within caecum as well as pellet formation but delayed gastric emptying and intestinal emptying when compared to DMSO-treated mice (Supplementary Figures S2A–E and Supplementary Table S2).

**FIGURE 1:**
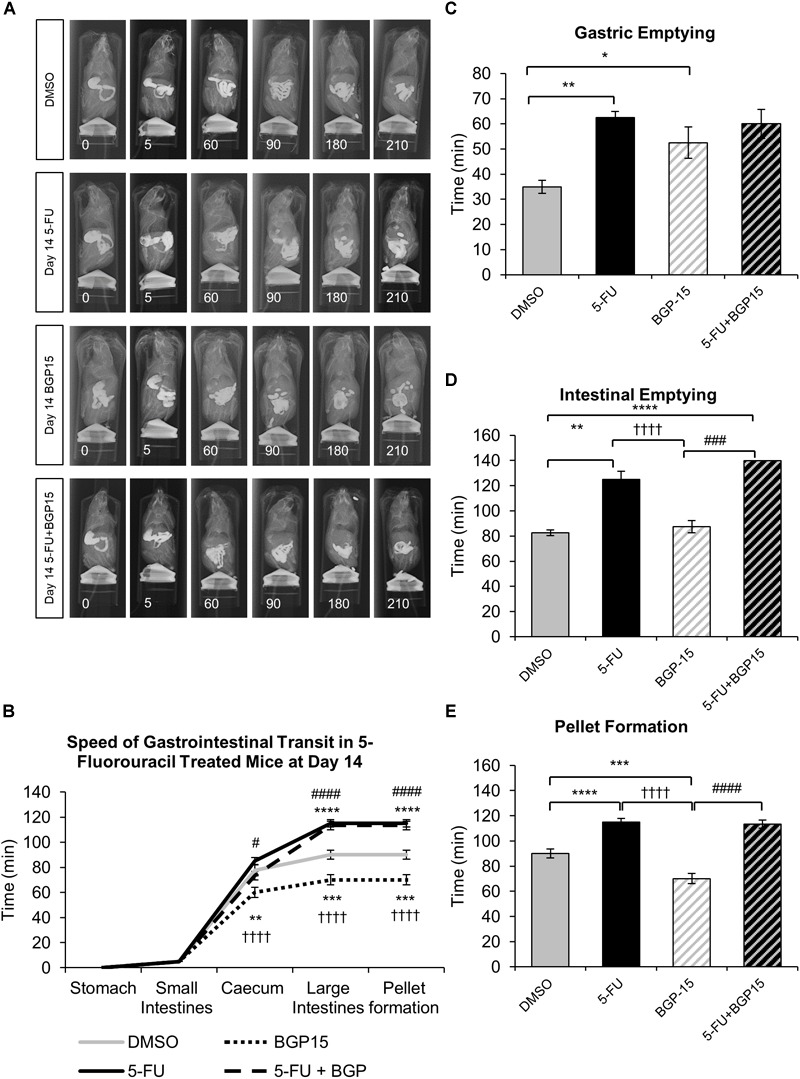
Gastrointestinal transit time, gastric, and intestinal emptying following repeated *in vivo* 5-FU ± BGP-15 administration at day 14. Representative X-ray images obtained from mice 5–210 min after intragastric barium sulfate (0.4 mL and 2.5 mg/mL) following 14 days of DMSO, 5-FU, BGP-15, and 5-FU+BGP-15 administration **(A)**. Time (min) taken for barium sulfate to reach the stomach, small intestines, caecum, and large intestines at 7 days following DMSO, 5-FU, BGP-15, and 5-FU+BGP-15 administration **(B)**. Time (min) taken for complete emptying of barium from the stomach **(C)**. Time (min) taken for complete emptying of barium from the small intestines **(D)**. Time (min) taken to form first pellet at 14 days following DMSO, 5-FU, BGP-15, and 5-FU+BGP-15 administration **(E)**. Data represented as mean ± SEM. ^∗^*P* < 0.05, ^∗∗^*P* < 0.01, ^∗∗∗^*P* < 0.001, ^∗∗∗∗^*P* < 0.0001 significantly different to DMSO. ^††††^*P* < 0.0001 significantly different to 5-FU. ^#^*P* < 0.05, ^###^*P* < 0.001, ^####^*P* < 0.0001 significantly different to BGP-15 (*n* = 5 mice/group).

Whilst BGP-15 appears to restore some level of normality to gastrointestinal transit at both day 3 (Supplementary Figure S1B) and 7 (Supplementary Figure S2B), when given in combination with 5-FU, BGP-15 treatment did not alleviate 5-FU induced gastrointestinal transit delays at day 14 (Figure 1A–E and Supplementary Table S3). After 14 days of 5-FU administration, movement of barium in the colon, gastric emptying, intestinal emptying and pellet formation were significantly delayed when compared to DMSO-treated mice (Figure 1A–E and Supplementary Table S3). No significant differences were found when comparing movement of barium within the caecum and colon, gastric emptying, intestinal emptying or pellet formation in 5-FU+BGP-15 to 5-FU-treated mice (Figure 1A–E and Supplementary Table S3). Treatment with BGP-15 alone sped up the movement of barium within the caecum as well as pellet formation when compared to DMSO-treated mice but delayed gastric emptying and had no effect on intestinal emptying (Figure 1A–E and Supplementary Table S3). When compared to 5-FU-treated mice, movement of barium in the caecum and colon, intestinal emptying and pellet formation was significantly faster in BGP-15-treated mice, while no significant difference in gastric emptying was found. Similar to 5-FU treatment, combined 5-FU+BGP-15 delayed the speed of barium movement in the colon and delayed the speed of pellet when compared to DMSO-treated mice (Figure 1A–E and Supplementary Table S3).

### Colonic Fecal Water Content Changes Following 5-FU ± BGP-15 Administration

To further define the clinical symptoms resulting from 5-FU ± BGP-15 administration, fresh fecal pellets were collected from all mice on days 3, 7, and 14 of treatment and dehydrated for 72 h. Fecal water content was subsequently calculated as the difference between wet and dry pellet weight and is presented as proportion of the total wet weight.

**FIGURE 2:**
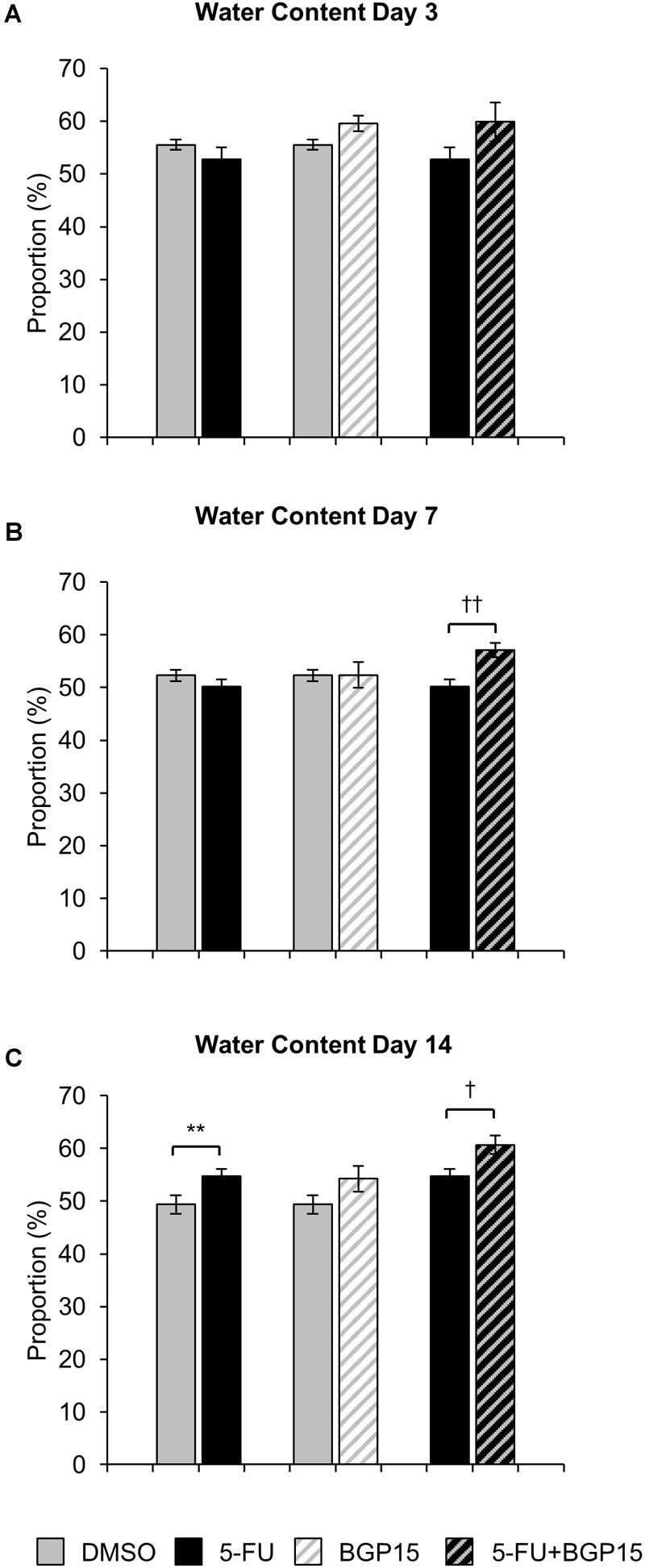
Water content following *in vivo* 5-FU ± BGP-15 administration. Fecal water content calculated as the difference between the wet weight and dry weight following 3 days **(A)**, 7 days **(B)**, and 14 days **(C)** treatment with DMSO, 5-FU, BGP-15, and 5-FU+BGP-15 treatment. Data represented as mean ± SEM. ^∗∗^*P* < 0.01 significantly different to DMSO group. ^†^*P* < 0.05, ^††^*P* < 0.01, significantly different to 5-FU (*n* = 5 mice/group).

No significant difference in fecal water content were found at day 3 or day 7 when comparing 5-FU to DMSO-treated mice (Figure 2A,B and Supplementary Table S4), however, at day 14 fecal water content was significantly increased in 5-FU-treated mice when compared to DMSO (*P* < 0.01, Figure 2C and Supplementary Table S4). Treatment with BGP-15 alone had no effect on fecal water content at any time point when compared to either DMSO or 5-FU-treated mice (Figure 2A–C and Supplementary Table S4). When given in combination with 5-FU, BGP-15 significantly increased fecal water content at day 7 (*P* < 0.01) and day 14 (*P* < 0.05) when compared to 5-FU-treated mice but had no effect at day 3 (Figure 2 and Supplementary Table S4).

### BGP-15 Exacerbates 5-FU-Induced Intestinal Inflammation

**FIGURE 3:**
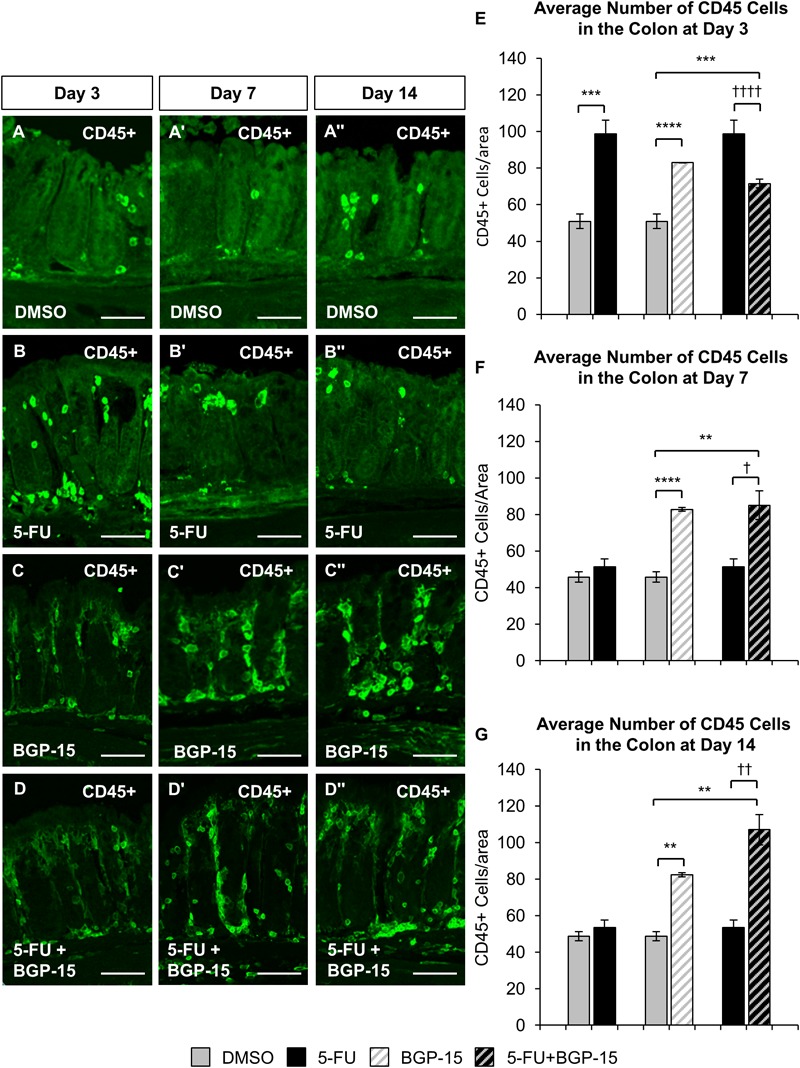
Inflammation in the colon repeated *in vivo* 5-FU ± BGP-15 administration. Cross sections of the colon labeled with antibody against CD45+ leukocytes (green) following 3, 7, and 14 days of DMSO **(A–A”)**, 5-FU **(B–B”)**, BGP-15 **(C–C”)**, and 5-FU+BGP-15 **(D–D”)** treatment, scale bar = 50 μm. Average number of CD45+ cells in the colon was counted per 2 mm^2^ at 3 days **(E)**, 7 days **(F)**, and 14 days **(G)** in DMSO, 5-FU, BGP-15, and 5-FU+BGP-15-treated mice. Data represented as mean ± SEM. ^∗∗^*P* < 0.01, ^∗∗∗^*P* < 0.001, ^∗∗∗∗^*P* < 0.0001 significantly different to DMSO, ^†^*P* < 0.05, ^††^*P* < 0.01, ^††††^*P* < 0.0001 significantly different to 5-FU (*n* = 5 mice/group).

To investigate the effects of BGP-15 on 5-FU-induced colonic inflammation, immune cell infiltration in the colon was analyzed (Figure 3). Immune cells in colonic cross sections were labeled with a pan leukocyte marker anti-CD45 antibody (Supplementary Figure S3) following 3, 7, and 14 days of DMSO (Figure 3A–A”), 5-FU (Figure 3B–B”), BGP-15 (Figure 3C–C”), and 5-FU+BGP-15 treatments (Figure 3D–D”). Total numbers of CD45 positive cells were counted within a 2 mm^2^ area.

A significant increase in the number of CD45 positive cells was found in the colon following 3 days of 5-FU administration (98 ± 7 cells/2 mm^2^, *P* < 0.001) when compared to DMSO-treated mice (51 ± 4 cells/2 mm^2^) (Figure 3E). BGP-15 treatment alone significantly increased the number of CD45 positive cells in the colon when compared to DMSO-treated mice at day 3 (83 ± 1 cells/2 mm^2^, *P* < 0.0001) (Figure 3E). When given with 5-FU, BGP-15 decreased the number of CD45 cells in the colon at day 3 (72 ± 2 cells/2 mm^2^) when compared to 5-FU-treated mice (*P* < 0.0001), but this was still significantly greater than in DMSO (*P* < 0.001) (Figure 3E).

The number of CD45 positive cells was unchanged after 7 (51 ± 4 cells/2 mm^2^) and 14 (53 ± 4 cells/2 mm^2^) days of 5-FU treatment compared to DMSO (day 7 DMSO: 46 ± 3 cells/2 mm^2^, day 14 DMSO: 49 ± 3 cells/2 mm^2^) (Figure 3F,G). BGP-15 treatment alone significantly increased the number of CD45 positive cells in the colon at day 7 (83 ± 1 cells/2 mm^2^, *P* < 0.0001) and 14 (82 ± 1 cells/2 mm^2^, *P* < 0.01) (Figure 3F,G). When given with 5-FU, BGP-15 significantly increased the number of CD45 positive cells at day 7 (85 ± 8 cells/2 mm^2^) compared to both DMSO-treated (*P* < 0.01) and 5-FU-treated mice (*P* < 0.05) (Figure 3F,G). Similarly, this treatment significantly increased the number of CD45 positive cells at day 14 (107 ± 8 cells/2 mm^2^), compared to both DMSO-treated (*P* < 0.01) and 5-FU-treated mice (*P* < 0.01) (Figure 3F,G).

### Co-administration of BGP-15 With 5-FU Exacerbates 5-FU-Induced Colonic Dysmotility

**FIGURE 4:**
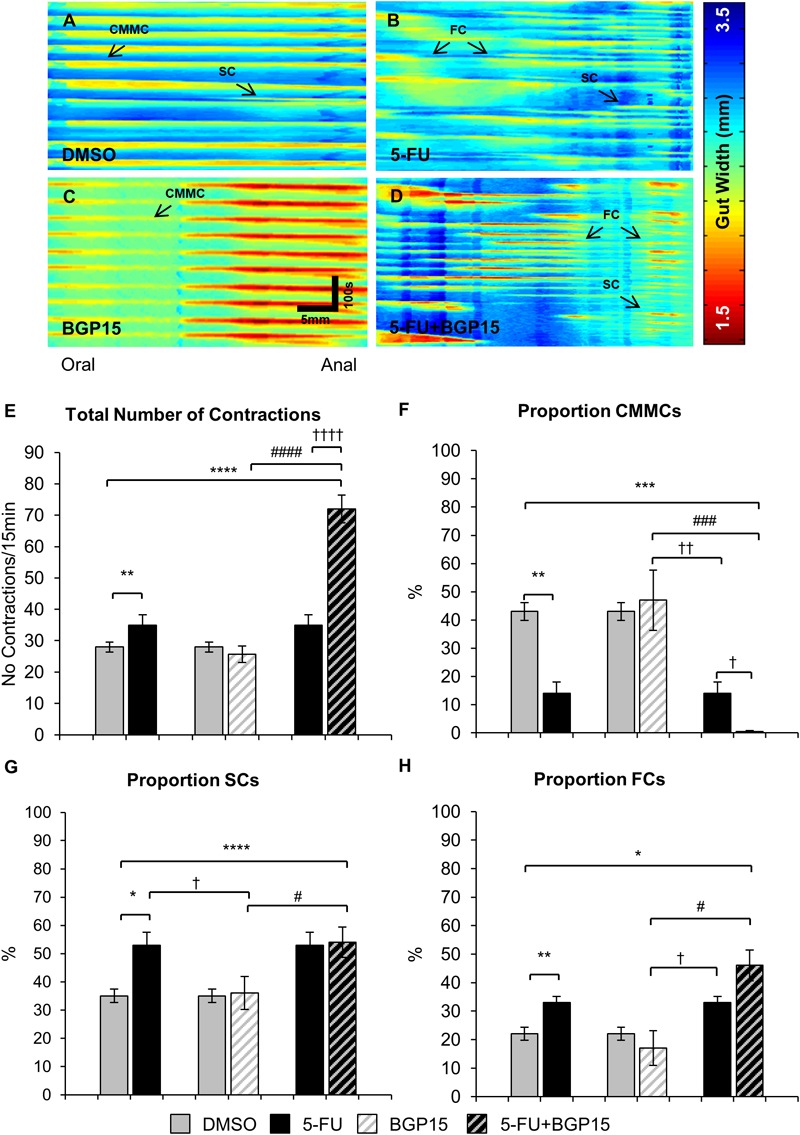
Colonic motility following repeated *in vivo* 5-FU ± BGP-15 administration. Representative spatiotemporal maps generated from digital video recordings of colonic motility following 14 days of DMSO **(A)**, 5-FU **(B)**, BGP-15 **(C)**, and 5-FU+BGP-15 **(D)** administration. Each contraction can be seen as a reduction in the gut width (red), while relaxation as an increase in the gut width (blue). Colonic migrating motor complexes (CMMCs) propagate >50% of the colon length, short contractions (SCs) propagate <50% of the colon length and fragmented contractions (FCs) are interrupted by period(s) of relaxation during contraction. Total number of contractions including all types of contractile activity in the colons from DMSO, 5-FU, BGP-15, and 5-FU+BGP-15 administration **(E)**. The proportion of CMMCs to the total number of contractions **(F)**. The proportion of SCs to the total number of contractions **(G)**. The proportion of FCs to the total number of contractions **(H)**. Data represented as mean ± SEM. ^∗^*P* < 0.05, ^∗∗^*P* < 0.01, ^∗∗∗^*P* < 0.001, ^∗∗∗∗^*P* < 0.0001, significantly different to DMSO group. ^†^*P* < 0.05, ^††^*P* < 0.01, ^††††^*P* < 0.0001, significantly different to 5-FU group. ^#^*P* < 0.05, ^###^*P* < 0.001, ^####^*P* < 0.0001, significantly different to BGP-15 (*n* = 5 mice/group).

To investigate effects of BGP-15 co-treatment on colonic motility, excised colons (*n* = 5 mice/group) were studied in organ bath experiments at day 14 of 5-FU treatment with and without BGP-15. Analysis of spatiotemporal maps from 5-FU-treated mice (Figure 4A–D) showed a significant increase in the total number of contractions compared to DMSO-treated mice (Figure 4E and Supplementary Table S5). BGP-15 treatment alone did not affect the total number of contractions compared to DMSO-treated mice (Figure 4E and Supplementary Table S5). When BGP-15 was given in combination with 5-FU, the total number of contractions was further increased when compared to DMSO, BGP-15 and 5-FU-treated mice (Figure 4E and Supplementary Table S5).

**FIGURE 5:**
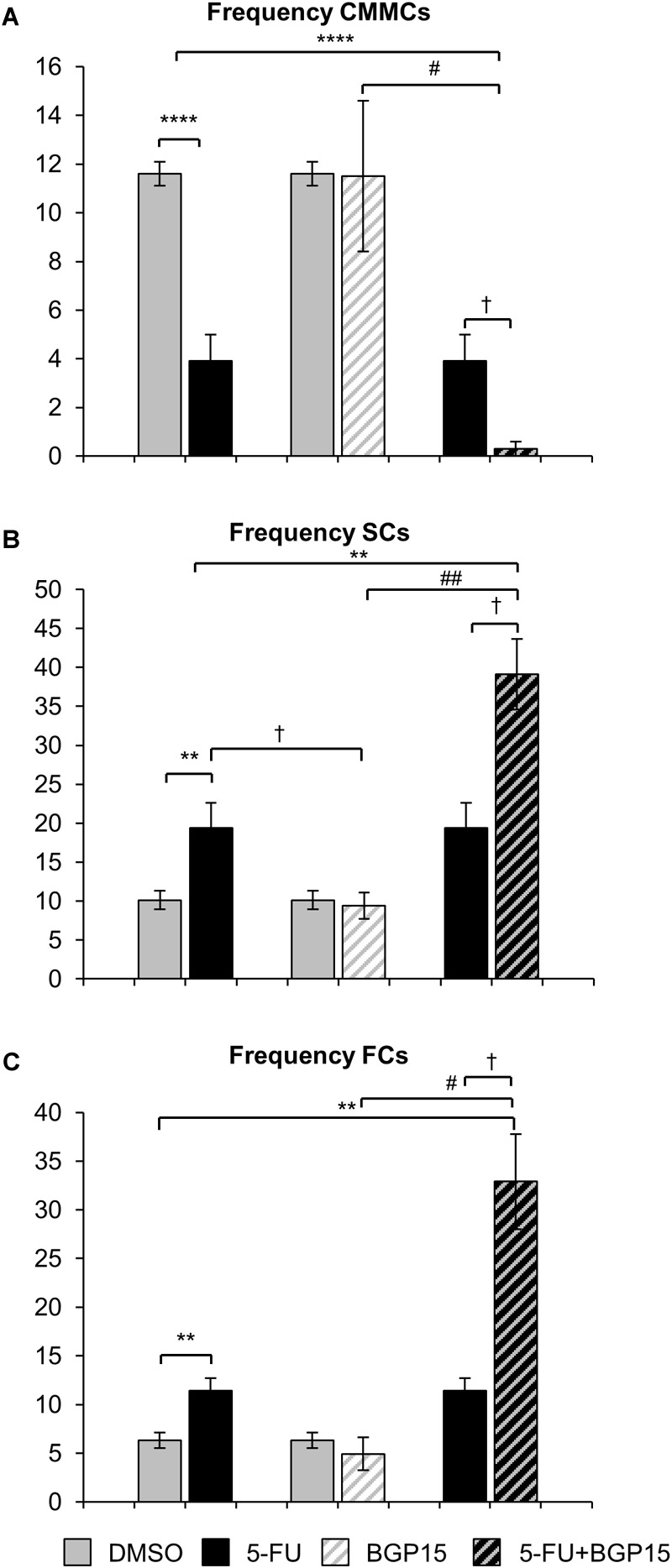
Frequency of different contractions following repeated *in vivo* 5-FU ± BGP-15 administration. Number of CMMCs per 15 min in the colons from DMSO, 5-FU, BGP-15, and 5-FU+BGP-15 administration **(A)**. Number of SCs per 15 min in the colons from DMSO, 5-FU, BGP-15, and 5-FU+BGP-15 administration **(B)**. Number of FCs per 15 min in the colons from DMSO, 5-FU, BGP-15, and 5-FU+BGP-15 administration **(C)**. Data represented as mean ± SEM. ^∗∗^*P* < 0.01, ^∗∗∗∗^*P* < 0.0001 significantly different to DMSO group. ^†^*P* < 0.05 significantly different to 5-FU group. ^#^*P* < 0.05, ^##^*P* < 0.01 significantly different to BGP-15 (*n* = 5 mice/group).

Treatment with 5-FU significantly decreased the proportion and frequency of CMMCs when compared to DMSO-treated mice (Figure 4F, 5A and Supplementary Table S5). BGP-15 treatment alone did not alter the proportion or frequency of CMMCs from DMSO (Figure 4F, 5A and Supplementary Table S5). When BGP-15 was given with 5-FU, it further reduced the proportion and frequency of CMMCs when compared to DMSO, BGP-15, and 5-FU-treated mice (Figure 4F, 5A and Supplementary Table S5).

Treatment with 5-FU significantly increased the proportion or frequency of SCs when compared to DMSO-treated mice (Figure 4G, 5B and Supplementary Table S5). BGP-15 treatment alone did not alter the proportion or frequency of SCs from DMSO (Figure 4G, 5B and Supplementary Table S5). 5-FU+BGP-15 treatment significantly increased both the proportion and frequency of SCs when compared to DMSO and BGP-15 treated mice (Figure 4G, 5B and Supplementary Table S5). When compared to 5-FU-treated mice, 5-FU+BGP-15 treatment further increased the frequency of SCs, but the proportion of SCs was unchanged (Figure 4G, 5B and Supplementary Table S5).

Treatment with 5-FU significantly increased the proportion and frequency of FCs when compared to DMSO-treated mice (Figure 4H, 5C and Supplementary Table S5). BGP-15 treatment alone did not alter the proportion or frequency of FCs from DMSO (Figure 4H, 5C and Supplementary Table S5). 5-FU+BGP-15 significantly increased the proportion and frequency of FCs compared to DMSO-treated mice (Figure 4H, 5C and Supplementary Table S5) and further significantly increased the frequency of FCs, but not the proportion of FCs compared to 5-FU alone (Figure 4H, 5C and Supplementary Table S5).

### Co-administration of BGP-15 With 5-FU Increases MitoSOX Fluorescence and Cytochrome *c* Release in the Myenteric Plexus

**FIGURE 6:**
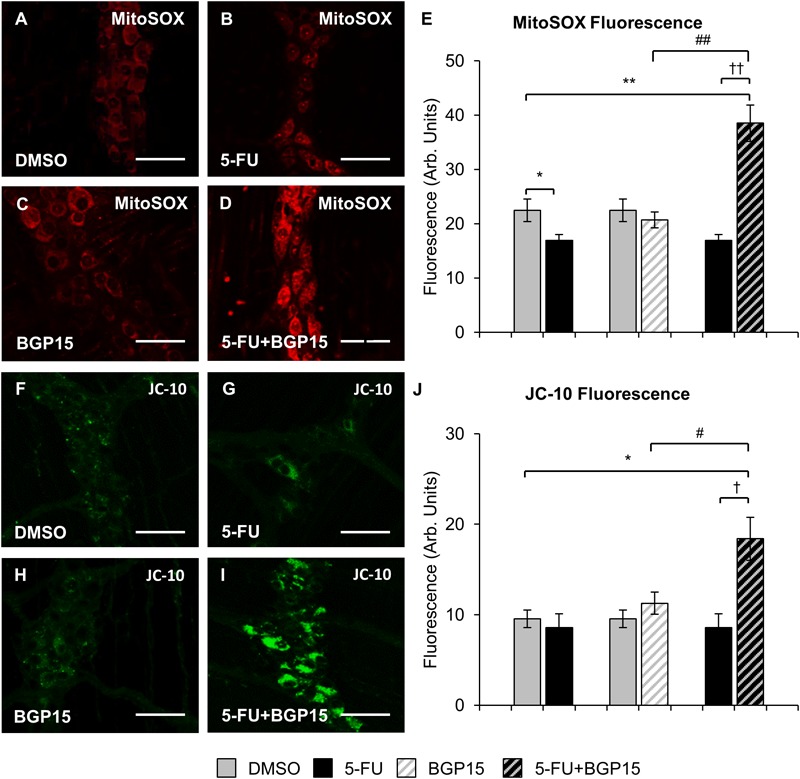
Mitochondrial superoxide and mitochondrial membrane potential in the myenteric plexus following repeated *in vivo* 5-FU ± BGP-15 administration. Fluorescent wholemount preparations of the myenteric plexus labeled with MitoSOX^TM^ Red M36008 in the colons from DMSO **(A)**, 5-FU **(B)**, BGP-15 **(C)**, and 5-FU+BGP-15-treated mice **(D)**, scale bar = 50 μm. Quantification of the levels of MitoSOX^TM^ Red M36008 production visualized by fluorescent probe in the myenteric plexus in colonic preparations from DMSO, BGP-15, 5-FU, and 5-FU+BGP-15-treated mice **(E)**. Fluorescent wholemount preparations of the myenteric plexus labeled with monomeric JC-10 in the colons from DMSO **(F)**, 5-FU **(G)**, BGP-15 **(H)**, and 5-FU+BGP-15-treated mice **(I)**, scale bar = 50 μm. Quantification of the levels of monomeric JC-10 production visualized by fluorescent probe in the myenteric plexus in colonic preparations from DMSO, BGP-15, 5-FU, and 5-FU+BGP-15-treated mice **(J)**. Data represented as mean ± SEM significantly different to 5-FU group. Data represented as mean ± SEM. ^∗^*P* < 0.05, ^∗∗^*P* < 0.01 significantly different to DMSO group. ^†^*P* < 0.05, ^††^*P* < 0.01 significantly different to 5-FU group. ^#^*P* < 0.05, ^##^*P* < 0.01 significantly different to BGP-15 (*n* = 5 mice/group).

To evaluate production of ROS following long-term 5-FU ± BGP-15 treatment, the myenteric plexus in colon samples collected at day 14 were probed with a fluorescent mitochondrial superoxide marker MitoSOX^TM^ Red M36008 (*n* = 5 mice/group) (Figure 6A–D). Decreased MitoSOX fluorescence was found in the myenteric plexus of the distal colon from 5-FU-treated mice (16.9 ± 1.08 arbitrary units) compared to DMSO-treated animals (22.5 ± 2.08 arbitrary units, *P* < 0.05) (Figure 6E). Treatment with BGP-15 alone (20.7 ± 1.47 arbitrary units) had no effect on MitoSOX fluorescence when compared to DMSO-treated mice (Figure 6E). When given with 5-FU, BGP-15 significantly increased MitoSOX fluorescence (38.5 ± 3.33 arbitrary units) when compared to DMSO-treated, BGP-15-treated and 5-FU-treated mice (*P* < 0.01 for all) (Figure 6E).

Diffusion of cytochrome *c* out of the mitochondria in the myenteric plexus as a result of mitochondrial membrane depolarization and increased permeability can be measured via fluorescence (green) of monomeric JC-10. To evaluate production of monomeric JC-10 following long-term 5-FU ± BGP-15 treatment, colon samples collected at day 14 were probed with the fluorescent marker JC-10 (*n* = 5 mice/group) (Figure 6F–I). No significant difference in JC-10 fluorescence was found in the myenteric plexus of the distal colon from 5-FU-treated mice (8.6 ± 1.51 arbitrary units) compared to DMSO-treated animals (9.5 ± 0.96 arbitrary units) (Figure 6J). Treatment with BGP-15 alone (11.3 ± 1.24 arbitrary units) had no effect on JC-10 fluorescence when compared to DMSO-treated mice (Figure 6J). When given with 5-FU, BGP-15 significantly increased JC-10 fluorescence (18.4 ± 2.38 arbitrary units) when compared to each of DMSO, BGP and 5-FU-treated mice (*P* < 0.05 for all) (Figure 6J).

### Co-treatment of BGP-15 With 5-FU Does Not Attenuate 5-FU-Induced Enteric Neuronal Loss, but Causes Changes in Cholinergic and Nitrergic Neuronal Populations

To investigate any changes to the total number of myenteric neurons, wholemount preparations of day 14 colon were labeled with a pan neuronal marker anti-βTub III for neuronal cell counting within a 2 mm^2^ area in DMSO, 5-FU, BGP-15, and 5-FU+BGP-15-treated mice (*n* = 5 mice/group).

Repeated *in vivo* administration of 5-FU induced myenteric neuronal loss (5-FU 1073 ± 19 neurons/area; DMSO 1221 ± 40 neurons/area, *P* < 0.0001). BGP-15 alone did not affect the number of myenteric neurons (1214 ± 41 neurons/area) compared to DMSO. When given with 5-FU, BGP-15 had no effect on the number of myenteric neurons (1076 ± 60 neurons/area) when compared to 5-FU-treated mice, which remained significantly reduced compared to DMSO-treated mice (*P* < 0.0001).

**FIGURE 7:**
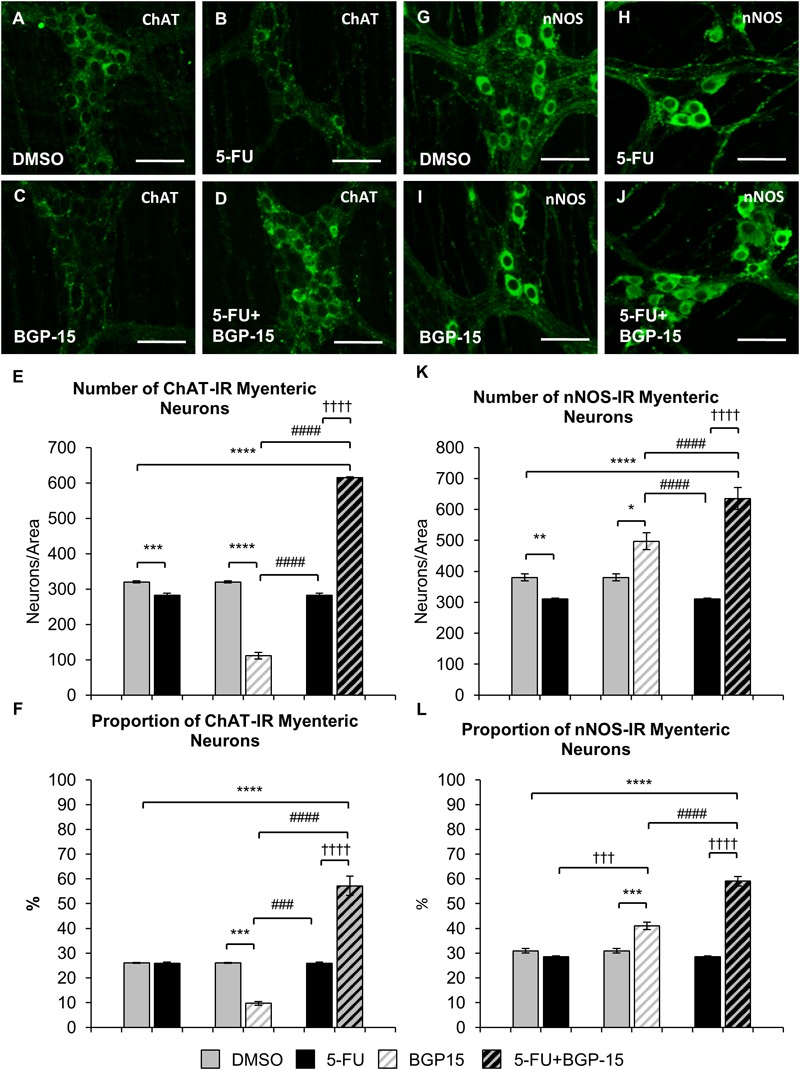
Effect of repeated *in vivo* 5-FU ± BGP-15 administration on average number and proportion of ChAT and nNOS-IR myenteric neurons. Wholemount preparations of ChAT-IR myenteric neurons (green) in the myenteric plexus of the distal colon in DMSO **(A)**, 5-FU **(B)**, BGP-15 **(C)**, and 5-FU+BGP-15-treated **(D)** mice, scale bar = 50 μm. Average number of ChAT-IR neurons in the colon was counted per 2 mm^2^ at 14 days in DMSO, 5-FU, BGP-15, and 5-FU+BGP-15-treated mice **(E)**. Proportion of ChAT-IR neurons to the total number of βTub III-IR myenteric neurons in the colon was counted at 14 days in DMSO, BGP-15, 5-FU, and 5-FU+BGP-15-treated mice **(F)**. Wholemount preparations of nNOS-IR neurons (green) in the myenteric plexus of the distal colon in DMSO **(G)**, 5-FU **(H)**, BGP-15 **(I)**, and 5-FU+BGP-15-treated **(J)** mice, scale bar = 50 μm. Average number of nNOS-IR neurons in the colon was counted per 2 mm^2^ at 14 days in DMSO, 5-FU, BGP-15, and 5-FU+BGP-15-treated mice **(K)**. Proportion of nNOS-IR neurons to the total number of βTub III-IR myenteric neurons in the colon was counted at 14 days in DMSO, 5-FU, BGP-15, and 5-FU+BGP-15-treated mice **(L)**. Data represented as mean ± SEM. ^∗^*P* < 0.05, ^∗∗^*P* < 0.01, ^∗∗∗^*P* < 0.001, ^∗∗∗∗^*P* < 0.0001, significantly different to DMSO group. ^†††^*P* < 0.001, ^††††^*P* < 0.0001, significantly different to 5-FU group. ^###^*P* < 0.001, ^####^*P* < 0.0001, significantly different to BGP-15 (*n* = 5 mice/group).

To determine if BGP-15 co-treatment was associated with changes in specific subpopulations of myenteric neurons, the average number and proportion of ChAT-IR and nNOS-IR motor and interneurons were analyzed in the colon from DMSO, 5-FU, BGP-15, and 5-FU+BGP-15-treated mice (*n* = 5 mice/group) (Figure 7A–D).

The number of ChAT-IR neurons in 5-FU-treated mice was decreased (286 ± 6 neurons/area) when compared to DMSO-treated mice (313 ± 4 neurons/area, *P* < 0.001) (Figure 7E). However, when the proportion of ChAT-IR neurons to the total number of cells was analyzed, no difference in the proportion of ChAT-IR neurons was found in 5-FU-treated mice (27 ± 0.6%) compared to DMSO-treated mice (25 ± 0.1%) (Figure 7F). Treatment with BGP-15 alone significantly decreased the number (137 ± 2 neurons/area) of ChAT-IR neurons compared to DMSO and 5-FU treated mice (*P* < 0.0001 for both), and the proportion (11 ± 0.4%) of ChAT-IR neurons when compared to both DMSO-treated mice and 5-FU-treated mice (Figure 7E, *P* < 0.001 for both). When given in combination with 5-FU, BGP-15 significantly increased the number (615 ± 2 neurons/area) and proportion (57 ± 4%) of ChAT-IR neurons compared to DMSO, BGP-15, and 5-FU-treated mice (*P* < 0.0001 for all) (Figure 7E,F).

The number of nNOS-IR neurons in 5-FU-treated mice was significantly decreased (311 ± 6) when compared to DMSO-treated mice (391 ± 9 neurons/area, *P* < 0.01, *n* = 5 mice/group) (Figure 7G,H,K). However, no difference in the proportion of nNOS-IR neurons was found when comparing 5-FU-treated (29 ± 0.4%) to DMSO-treated mice (32 ± 1%) (Figure 7L). Treatment with BGP-15 alone significantly increased the number of nNOS-IR neurons (497 ± 27 neurons/area) when compared to both DMSO-treated mice (*P* < 0.05) and 5-FU-treated mice (*P* < 0.0001) as well as the proportion of nNOS-IR neurons (41 ± 1.5%) when compared to both DMSO-treated mice and 5-FU-treated mice (*P* < 0.001 for both) (Figure 7L). When given with 5-FU, BGP-15 significantly increased the number (636 ± 35 neurons/area) and proportion (59 ± 2%) of nNOS-IR neurons compared to DMSO-treated, BGP-15-treated and 5-FU-treated mice (*P* < 0.0001 for all) (Figure 7I–L).

## Discussion

In this study, the effect of BGP-15 and 5-FU co-treatment on myenteric neurons and gastrointestinal functions was examined. Results indicate that BGP-15 co-treatment increased mitochondrial superoxide production and mitochondrial depolarization in the myenteric plexus increased the number and proportion of ChAT-IR and nNOS-IR neurons in the myenteric plexus. BGP-15 co-treatment failed to protect against 5-FU-induced neuronal loss and significantly increased the inflammation in colonic cross sections, as measured by the presence of CD45 positive cells. Although BGP-15 co-treatment delayed the onset of gastrointestinal symptoms at days 3 and 7, it had no effect on 5-FU-induced changes in gastrointestinal transit speed, gastric emptying, intestinal emptying or pellet formation at the end of treatment. BGP-15 co-treatment exacerbated 5-FU-induced colonic dysmotility at day 14 by reducing the number and proportion of CMMCs and increasing the number and proportion of FCs. These changes were correlated with increased fecal water content, which may be indicative of diarrhea.

Results of the current study have indicated that BGP-15 co-treatment had no beneficial effect on 5-FU-induced delays in gastrointestinal transit, gastric emptying, intestinal emptying, or pellet formation at end of treatment, and is not an appropriate neuroprotective agent when given with 5-FU. This is in contrast to our previous findings where co-treatment of BGP-15 with oxaliplatin improved oxaliplatin-induced delays in gastrointestinal transit, intestinal emptying, and pellet formation (McQuade et al., 2018). These conflicting results may, in part, be a consequence of exacerbation of inflammation experienced during 5-FU administration (McQuade et al., 2016b), and/or *in vivo* interactions between 5-FU and BGP-15.

The cytoprotective agent BGP-15 is a multi-target compound known to inhibit PARP 1. Although pharmacological PARP inhibition has yielded promising results for neuroprotection in animal models of ischemia, traumatic brain injury, diabetic neuropathy, chemotherapy-induced peripheral neuropathy (Plaschke et al., 2000; LaPlaca et al., 2001; Obrosova et al., 2008; Lupachyk et al., 2011; Brederson et al., 2012; Ta et al., 2013), findings from the current study demonstrate that BGP-15 treatment alone and in combination with 5-FU induces colonic inflammation at all time points. PARP1 regulates the expression of various proteins associated with inflammation at the transcriptional level, including iNOS, intercellular adhesion molecule-1 and major histocompatibility complex class II via its activity on NF-κB (Hiromatsu et al., 1992; Pacher and Szabo, 2008; Peralta-Leal et al., 2009). Whilst there is some contention as to the role of PARP in inflammation, at a cellular level, PARP1 has been implicated in the activation and progression of inflammation (Ba and Garg, 2011). Reduction of functional PARP1 has been found to decrease the expression of several pro-inflammatory mediators, including cytokines, chemokines, adhesion molecules, and enzymes (Peralta-Leal et al., 2009). Furthermore, it has also been reported that PARP1 actively regulates other transcription factors implicated in cellular stress and inflammation, including AP-1, OCT-1, SP-1, HIF, and STAT1 (Peralta-Leal et al., 2009). Given the vast and varied roles of PARP in inflammatory mediation, inhibition of PARP by BGP-15 may contribute to the detrimental effects of BGP-15 treatment in our study.

The results of the current study demonstrate that inflammation following combined 5-FU+BGP-15 treatment, but not 5-FU treatment alone, is associated with increased mitochondrial superoxide production in the myenteric plexus observed at day 14. It is well known that inflammatory cells liberate a number of ROS at the site of inflammation leading to exaggerated oxidative stress (Biswas, 2016), whilst ROS can initiate intracellular signaling cascades that enhance pro-inflammatory gene expression (Anderson et al., 1994; Flohé et al., 1997). Although at physiological concentrations ROS may function as signaling molecules that regulate cell growth, cellular adhesion, differentiation, senescence, and apoptosis (Thannickal and Fanburg, 2000; Dröge, 2002), chronic or prolonged ROS production is central to the progression of inflammatory conditions (Mittal et al., 2014). The intricate balance between cell death and cell survival is largely modulated by intracellular ROS generation (Mittal et al., 2014).

Results of the current study have demonstrated that co-administration of BGP-15 with 5-FU, but not 5-FU alone, is associated with mitochondrial depolarization leading to cytochrome *c* release indicative of apoptotic cell death. This, in turn, was correlated with neuronal loss in 5-FU-treated mice that was not alleviated by co-treatment with BGP-15. While this is the first study to investigate the effect of BGP-15 in combination with 5-FU treatment on the myenteric plexus, our previous work investigating combined oxaliplatin and BGP-15 treatment has demonstrated that BGP-15 reduced markers of oxidative stress and improved neuronal survival at the level of the myenteric plexus (McQuade et al., 2018). The underlying mechanisms responsible for these opposing results is unknown but may be related to chemotherapeutic mechanism of action or *in vivo* drug interaction. Previous studies investigating the effects of a PARP inhibitor rucaparib in combination with 5-FU in the treatment of acute myeloid leukemia and acute lymphoblastic leukemia found that 5-FU and rucaparib synergize to induce a massive DNA damage *ex vivo*, measured by expression of phosphorylated histone (γH2AX) (Falzacappa et al., 2015). This “hyperdamage,” however, was not induced in PARP inhibition associated with administration of an oxaliplatin predecessor, platinum compound cisplatin (Falzacappa et al., 2015), suggesting specificity in the combination of PARP inhibition and 5-FU potentially resulting from *in vivo* drug interactions. The underlying mechanism responsible for the synergistic 5-FU+BGP-15 enteric neuronal damage in the current study is not known but may be due to loss of functional PARP in various pathways or to the way that 5-FU exerts its cytotoxicity, which is poorly understood.

We have previously shown that long-term 5-FU administration has no effect on the proportion of either ChAT or nNOS-IR neurons in the myenteric plexus (McQuade et al., 2016b). However, in the present study BGP-15 treatment alone and in combination with 5-FU significantly influenced the proportion of both ChAT and nNOS-IR neurons. Following BGP-15 treatment the proportion of nNOS-IR neurons was increased by 10% when compared to DMSO-treated mice, combined 5-FU+BGP-15 treatment further increased the proportion of nNOS-IR neurons by 28% when compared to 5-FU-treated mice. Whilst this increase in nNOS-IR neurons was accompanied by a concurrent loss in proportion of ChAT-IR neurons in BGP-15-treated mice (15% loss when compared to DMSO), interestingly in 5-FU+BGP-15-treated mice the proportion of ChAT-IR neurons was simultaneously increased alongside nNOS-IR neurons. These changes may imply: (1) that there is upregulation of ChAT and nNOS-IR in neurons that already express both, (2) that there has been an important change in neurochemical phenotype, or (3) both. It has been established that neurons can alter their chemical phenotype under pathological conditions (Sharkey and Kroese, 2001; Csillik et al., 2003; Ekblad and Bauer, 2004; Kaleczyc et al., 2007). Change in either neuropeptide expression or neurochemical phenotype could result in disrupted motility, due to effects on interneurons, intrinsic primary afferent neurons or motor neurons.

The proportion of ChAT-IR neurons in DMSO-treated mice reported in this study (26%) is lower than previously described in Balb/c mice (55%) (Sang and Young, 1998). Whilst, these discrepancies in the proportion of ChAT-IR neurons may be attributed to investigative and/or analytical errors, it has previously been noted that ChAT immunoreactivity is often centered in the cytoplasm with weak cell surface immunoreactivity, resulting in faint labeling across some ChAT-IR neurons (Furness et al., 2004). It is possible that ineffective labeling of ChAT has resulted in a lower than normal proportion of ChAT-IR neurons in the cohort of mice used in this study, however, the proportion of ChAT reported in this study is in line with our previously published work (McQuade et al., 2016b). Regardless, whether the increase in ChAT-IR in 5-FU+BGP-15-treated mice is associated with *de novo* synthesis of ChAT and what the exact functional phenotype of these neurons is requires further investigation. Further studies investigating the co-localization of nNOS and ChAT-IR neurons in 5-FU+BGP-15-treated mice need to be undertaken.

Loss of neurons in both 5-FU-treated and BGP-15 co-treated mice was associated with chronic colonic dysmotility. Moreover, co-treatment of BGP-15 with 5-FU exacerbated 5-FU-induced colonic dysmotility by reducing the number and proportion of CMMCs and increasing the number and proportion of FCs. Although this is in contrast to our previous data demonstrating that BGP-15 co-treatment with oxaliplatin alleviated oxaliplatin-induced colonic dysmotility (McQuade et al., 2018), the current findings are in line with our studies demonstrating that loss of enteric neurons negatively affects patterns of colonic motility (McQuade et al., 2016a, 2018). Our previous data indicate that 5-FU-induced myenteric neuronal loss of approximately 12% severely impacted patterns of colonic motility, significantly reducing the frequency and proportion of CMMCs whilst increasing frequency and proportion of SCs and FCs (McQuade et al., 2016a). In the current study the overall proportion of CMMCs was reduced by 93% in 5-FU+BGP-15-treated mice when compared to 5-FU-treated mice, whilst the proportion of SCs and FCs were increased by 86 and 175%, respectively. Short and FCs play a vital role in the construction of productive motor patterns in the healthy intestine (Gwynne et al., 2004). In the current study these altered patterns of colonic motility in 5-FU+BGP-15-treated mice were associated with increased fecal water content. Although pellet formation was delayed in 5-FU+BGP-15-treated mice, it is possible that mucosal damage and the consequential inflammatory response reduced the absorptive capacity in the colon. Furthermore, heightened cholinergic innervation may have contributed to increased intestinal secretion resulting in increased fecal water expulsion (Fung et al., 2018).

Although previously BGP-15, has shown cytoprotective potential (Racz et al., 2002; Bardos et al., 2003; Sarszegi et al., 2012) as well as chemo-potentiating capacity, in animal models of whole body radiation, death of PARP^-/-^ mice has been linked to gastrointestinal failure (Herceg and Wang, 2001). When compared to wild type controls all PARP^-/-^ mice died within 10 days of treatment with lethality associated with massive cell death and hemorrhage in small intestinal villi resulting in potential changes to intestinal absorption and sections and systemic dehydration in mice (Herceg and Wang, 2001). Moreover, PARP^-/-^ mice had severe hemorrhage in the glandular stomach (Masutani et al., 2000).

## Conclusion

In conclusion, this study is the first to demonstrate that BGP-15 exacerbated colonic inflammation and triggered the production of mitochondrial superoxide in 5-FU-treated mice. These changes were not beneficial to neuronal survival and exacerbated 5-FU-induced colonic dysfunction, resulting in increased fecal water content, indicative of diarrhea. Although PARP1 inhibition has shown both chemotherapeutic and neuroprotective potential in previous studies, it is clear from the work presented here that the effectiveness of BGP-15 application is heavily reliant on drug combination. Inflammation induced by 5-FU may also be a significant factor in estimating the prognostic outcome of BGP-15 application. Further studies need to be undertaken investigating the relevance of inflammation in mediating BGP-15 efficacy and understanding the relationship between oxidative stress and inflammation.

## Ethics Statement

All procedures were approved by the Victoria University Animal Experimentation Ethics Committee and performed in accordance with the guidelines of the National Health and Medical Research Council (NHMRC) Australian Code of Practice for the Care and Use of Animals for Scientific Purposes.

## Author Contributions

RM: conception and design, collection, analysis and interpretation of data, and manuscript writing. MAT: collection and analysis of data and manuscript writing. AP, RA, JB, and ER: interpretation of data and manuscript revision. KN: conception and design, interpretation of data, and manuscript revision. All authors approved the final version of the manuscript.

## Conflict of Interest Statement

The authors declare that the research was conducted in the absence of any commercial or financial relationships that could be construed as a potential conflict of interest.

## Supplementary Material

FIGURE S1 Gastrointestinal transit time, gastric and intestinal emptying following repeated *in vivo* 5-FU ± BGP-15 administration at day 3. Representative X-ray images obtained from mice 5–210 min after intragastric barium sulfate (0.4 mL and 2.5 mg/mL) administration following 3 days of DMSO, 5-FU, BGP-15, and 5-FU+BGP-15 administration **(A)**. Time (min) taken for barium sulfate to reach the stomach, small intestines, caecum, and large intestines in at 3 days following DMSO, 5-FU, BGP-15, and 5-FU+BGP-15 administration **(B)**. Time (min) taken for complete emptying of barium from the stomach **(C)**. Time (min) taken for complete emptying of barium from the small intestines **(D)**. Time (min) taken to form first pellet at 3 days following DMSO, 5-FU, BGP-15, and 5-FU+BGP-15 administration **(E)**. Data represented as mean ± SEM. ^∗^*P* < 0.05, ^∗∗^*P* < 0.01, ^∗∗∗^*P* < 0.001, ^∗∗∗∗^*P* < 0.0001 significantly different DMSO group. ^†^*P* < 0.05, ^††^*P* < 0.1, ^†††^*P* < 0.001, ^††††^*P* < 0.0001, significantly different to 5-FU. ^#^*P* < 0.05, significantly different to BGP-15 (*n* = 5 mice/group).

FIGURE S2 Gastrointestinal transit time, gastric, and intestinal emptying following repeated *in vivo* 5-FU ± BGP-15 administration at day 7. Representative X-ray images obtained from mice 5–210 min after intragastric barium sulfate (0.4 mL and 2.5 mg/mL) administration following 7 days of DMSO, 5-FU, BGP-15, and 5-FU+BGP-15 administration **(A)**. Time (min) taken for barium sulfate to reach the stomach, small intestines, caecum, and large intestines at 7 days following DMSO, 5-FU, BGP-15, and 5-FU+BGP-15 administration **(B)**. Time (min) taken for complete emptying of barium from the stomach **(C)**. Time (min) taken for complete emptying of barium from the small intestines **(D)**. Time (min) taken to form first pellet at 7 days following DMSO, 5-FU, BGP-15, and 5-FU+BGP-15 administration **(E)**. Data represented as mean ± SEM. ^∗^*P* < 0.05, ^∗∗∗^*P* < 0.001, ^∗∗∗∗^*P* < 0.0001, significantly different to DMSO group. ^†††^*P* < 0.001, significantly different to 5-FU. ^##^*P* < 0.01, ^####^*P* < 0.0001, significantly different to BGP-15 (*n* = 5 mice/group).

FIGURE S3 Negative control for immunolabeling with CD45 antibody. Labeling with Alexa-Fluor 488 resulted in no visible stain at baseline fluorescence **(A)**, at 50% maximum fluorescence labeling with Alexa-Fluor 488 resulted in some autofluorescence, however no discernible CD45+ cells were present **(A’)**. Representative slide of CD45+ labeling acquired at 50% maximum fluorescence for comparison **(B)**.

TABLE S1 Speed of transit and emptying following 3 days repeated *in vivo* 5-FU ± BGP-15 administration.

TABLE S2 Speed of transit and emptying following 7 days repeated *in vivo* 5-FU ± BGP-15 administration.

TABLE S3 Speed of transit and emptying following 14 days repeated *in vivo* 5-FU ± BGP-15 administration.

TABLE S4 Fecal water content following 14 days repeated *in vivo* 5-FU ± BGP-15 administration.

TABLE S5 Colonic motility following 14 days repeated *in vivo* 5-FU ± BGP-15 administration.

## Abbreviations

AP-1: activator protein-1
ChAT: choline acetyltransferase
CMMC: colonic migrating motor complex
CRC: colorectal cancer
DMSO: dimethyl sulfoxide
ENS: enteric nervous system
FC: fragmented contraction
FOLFIRI: 5-fluorouracil, irinotecan, and leucovorin
FOLFOX: 5-fluorouracil, oxaliplatin, and leucovorin
5-FU: 5-fluorouracil
GI: gastrointestinal
HIF: hypoxia-inducible factor
iNOS: inducible nitric oxide synthase
IR: immunoreactive
nNOS: neuronal nitric oxide synthase
NF-κB: nuclear factor-κB
NHMRC: National Health and Medical Research Council
NO: nitric oxide
OCT-1: organic cation transporter-1
OXL: oxaliplatin
PARP: poly(ADP)-ribose polymerase
ROS: reactive oxygen species
SC: short contraction
SNP: sodium nitroprusside
SP-1: specificity protein-1
STAT1: signal transducer and activator of transcription 1
VAChT: vesicular acetylcholine transferase
