# Supplementary figures and images for: Co-treatment With BGP-15 Exacerbates 5-Fluorouracil-Induced Gastrointestinal Dysfunction
Source: Front Neurosci. 2019 May 8;13:449. doi: 10.3389/fnins.2019.00449 (PMC6518025; doi:10.3389/fnins.2019.00449)

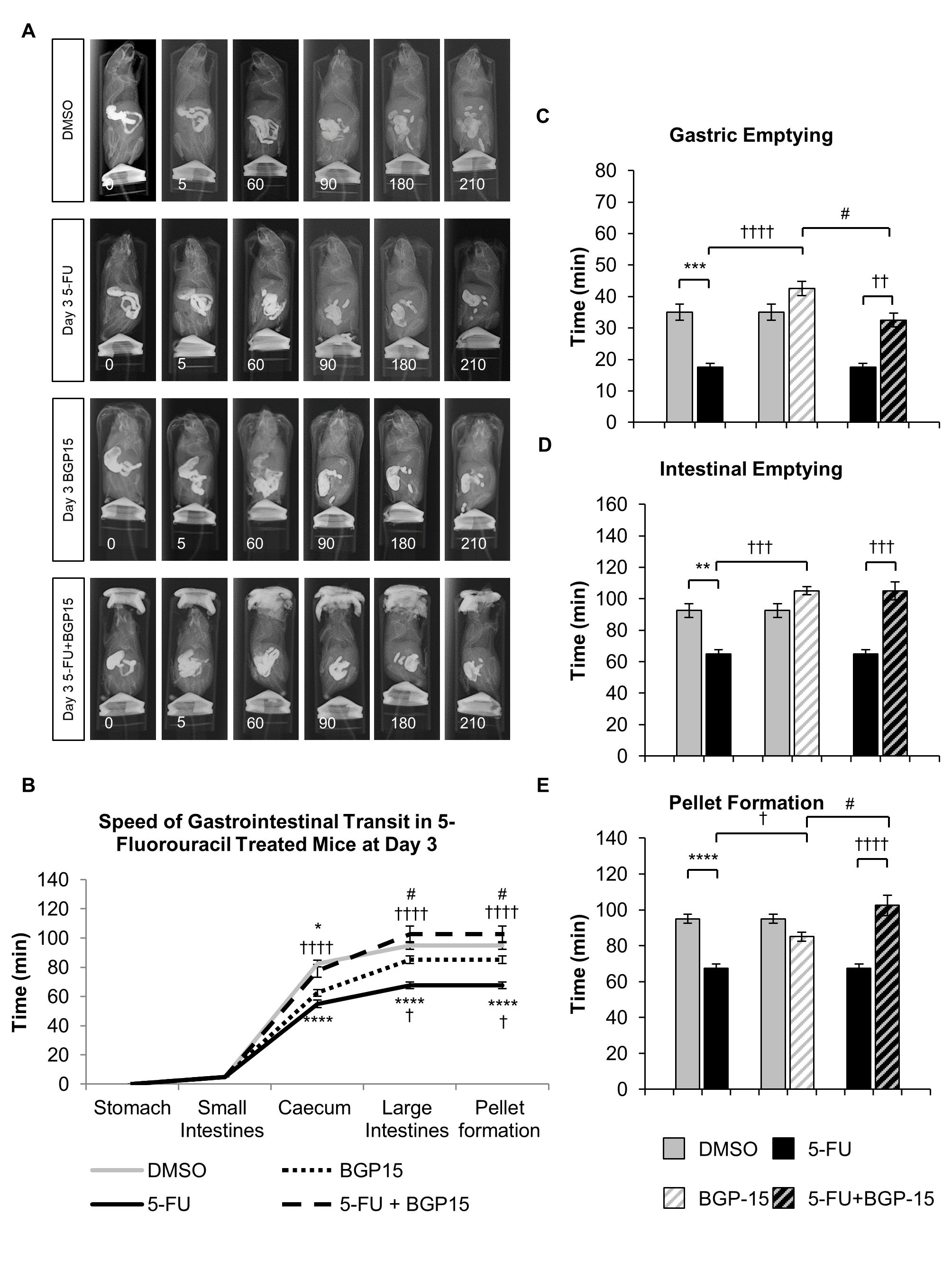

Supplement: FIGURE S1 — Gastrointestinal transit time, gastric and intestinal emptying following repeated in vivo 5-FU ± BGP-15 administration at day 3. Representative X-ray images obtained from mice 5–210 min after intragastric barium sulfate (0.4 mL and 2.5 mg/mL) administration following 3 days of DMSO, 5-FU, BGP-15, and 5-FU+BGP-15 administration (A). Time (min) taken for barium sulfate to reach the stomach, small intestines, caecum, and large intestines in at 3 days following DMSO, 5-FU, BGP-15, and 5-FU+BGP-15 administration (B). Time (min) taken for complete emptying of barium from the stomach (C). Time (min) taken for complete emptying of barium from the small intestines (D). Time (min) taken to form first pellet at 3 days following DMSO, 5-FU, BGP-15, and 5-FU+BGP-15 administration (E). Data represented as mean ± SEM. ∗P < 0.05, ∗∗P < 0.01, ∗∗∗P < 0.001, ∗∗∗∗P < 0.0001 significantly different DMSO group. †P < 0.05, ††P < 0.1, †††P < 0.001, ††††P < 0.0001, significantly different to 5-FU. #P < 0.05, significantly different to BGP-15 (n = 5 mice/group). [file Image_1.TIF]

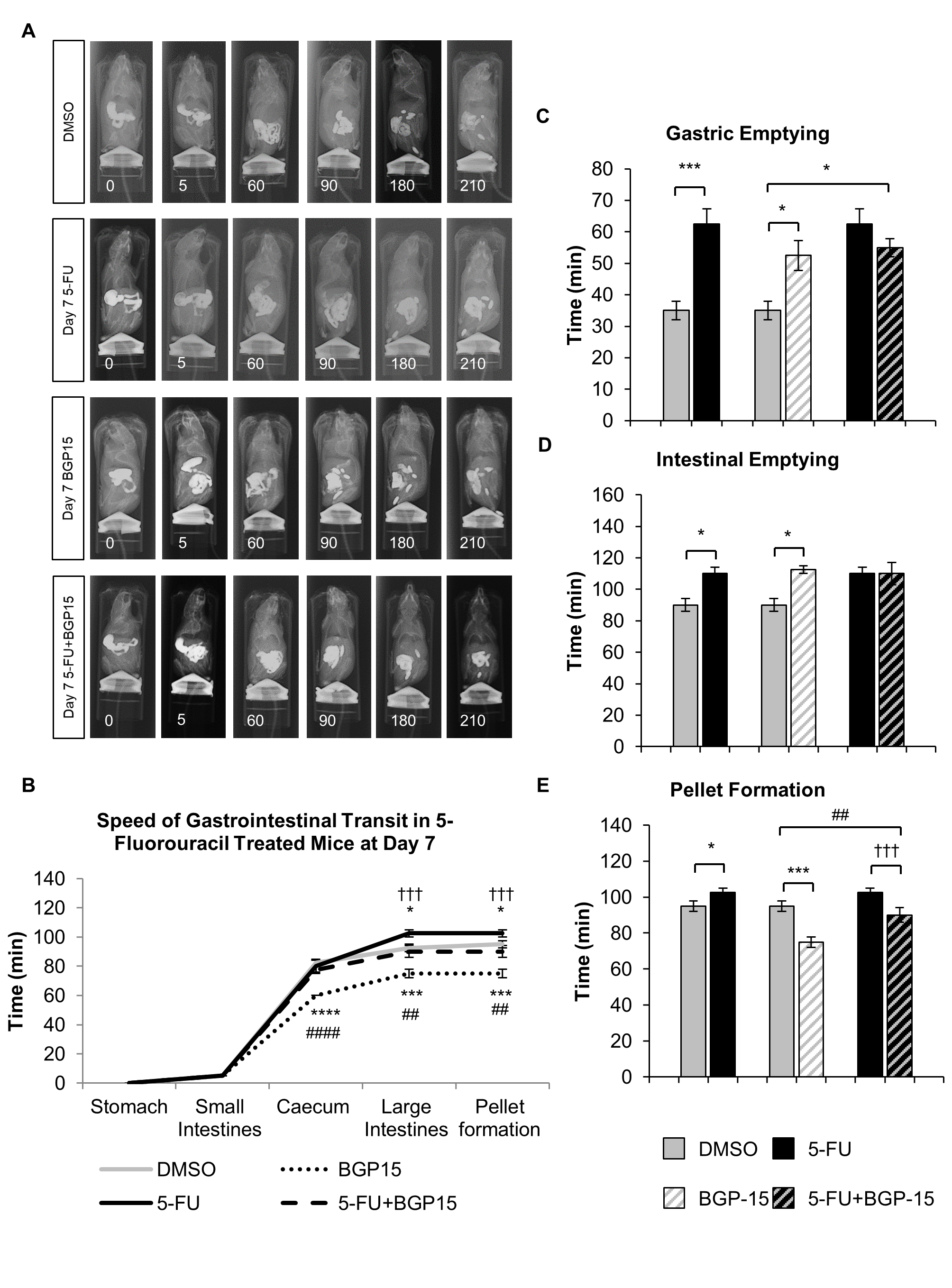

Supplement: FIGURE S2 — Gastrointestinal transit time, gastric, and intestinal emptying following repeated in vivo 5-FU ± BGP-15 administration at day 7. Representative X-ray images obtained from mice 5–210 min after intragastric barium sulfate (0.4 mL and 2.5 mg/mL) administration following 7 days of DMSO, 5-FU, BGP-15, and 5-FU+BGP-15 administration (A). Time (min) taken for barium sulfate to reach the stomach, small intestines, caecum, and large intestines at 7 days following DMSO, 5-FU, BGP-15, and 5-FU+BGP-15 administration (B). Time (min) taken for complete emptying of barium from the stomach (C). Time (min) taken for complete emptying of barium from the small intestines (D). Time (min) taken to form first pellet at 7 days following DMSO, 5-FU, BGP-15, and 5-FU+BGP-15 administration (E). Data represented as mean ± SEM. ∗P < 0.05, ∗∗∗P < 0.001, ∗∗∗∗P < 0.0001, significantly different to DMSO group. †††P < 0.001, significantly different to 5-FU. ##P < 0.01, ####P < 0.0001, significantly different to BGP-15 (n = 5 mice/group). [file Image_2.TIF]

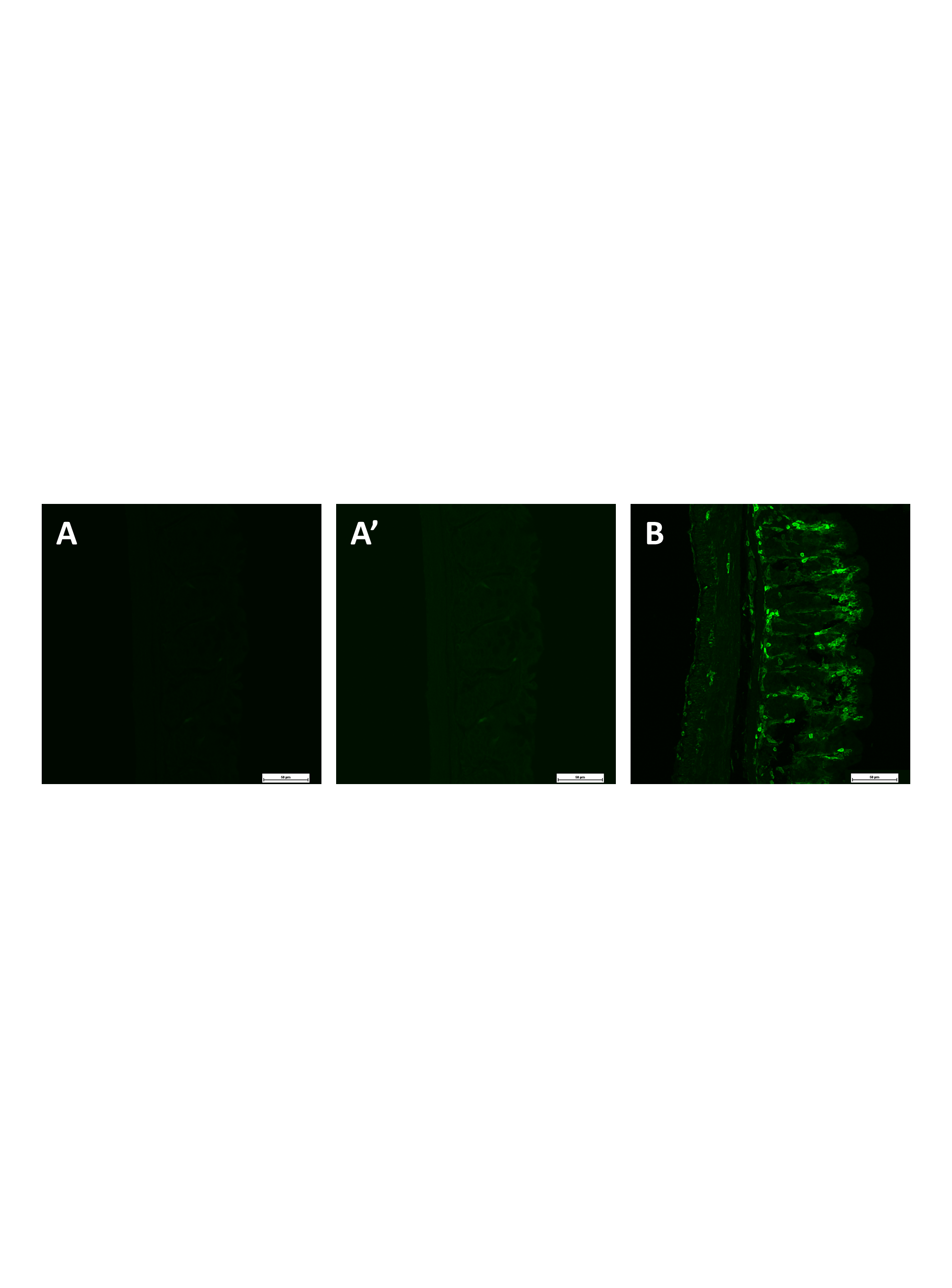

Supplement: FIGURE S3 — Negative control for immunolabeling with CD45 antibody. Labeling with Alexa-Fluor 488 resulted in no visible stain at baseline fluorescence (A), at 50% maximum fluorescence labeling with Alexa-Fluor 488 resulted in some autofluorescence, however no discernible CD45+ cells were present (A’). Representative slide of CD45+ labeling acquired at 50% maximum fluorescence for comparison (B). [file Image_3.TIF]
