# Supplementary material for: Co-treatment With BGP-15 Exacerbates 5-Fluorouracil-Induced Gastrointestinal Dysfunction
Source: Front Neurosci. 2019 May 8;13:449. doi: 10.3389/fnins.2019.00449 (PMC6518025; doi:10.3389/fnins.2019.00449)
Supplement: TABLE S1 — Speed of transit and emptying following 3 days repeated in vivo 5-FU ± BGP-15 administration. [file Table_1.DOCX]

**Supplementary Table S1 Speed of transit and emptying following 3 days repeated *in vivo* 5-FU+/-BGP-15 administration**

| **Parameters measured** | | **DMSO** | **5-FU** | **BGP-15** | **5-FU+BGP-15** |
| --- | --- | --- | --- | --- | --- |
| Speed of transit (time to reach each region, min) | **Stomach** | 0±0 | 0±0 | 0±0 | 0±0 |
|  | **Small Intestines** | 5±0 | 5±0 | 5±0 | 5±0 |
|  | **Caecum** | 82.5±3 | 55±3  **** | 62.5±3  * | 77.5±4  †††† |
|  | **Large Intestines** | 95±3 | 67.5±3  **** | 85±3  † | 102.5±6  ††††  # |
| Time for complete barium emptying (min) | **Gastric emptying** | 37±3 | 16±1  *** | 43±3  †††† | 33±2  ††  # |
|  | **Intestinal emptying** | 97±3 | 63±3  ** | 103±3  ††† | 105±6  ††† |
|  | **Pellet Formation** | 97±3 | 66±3  **** | 87±3  † | 103±6  ††††  # |

**P*<0.05, ***P*<0.01, ****P*<0.001, *****P*<0.0001 significantly different to DMSO-treated group. ^†^*P*<0.05, ^††^*P*<0.1, ^†††^*P*<0.001, ^††††^*P*<0.0001, significantly different to 5-FU-treated group. ^#^*P*<0.05, significantly different to BGP-15-treated group (n=5 mice/group).

**Supplementary Table S2 Speed of transit and emptying following 7 days repeated *in vivo* 5-FU+/-BGP-15 administration**

| **Parameters measured** | | **DMSO** | **5-FU** | **BGP-15** | **5-FU+BGP-15** |
| --- | --- | --- | --- | --- | --- |
| Speed of transit (time to reach each region, min) | **Stomach** | 0±0 | 0±0 | 0±0 | 0±0 |
|  | **Small Intestines** | 5±0 | 5±0 | 5±0 | 5±0 |
|  | **Caecum** | 83±3 | 80±5 | 60±0  **** | 78±3  #### |
|  | **Large Intestines** | 93±3 | 105±4  * | 78±3  *** | 90±4  †††  ## |
| Time for complete barium emptying (min) | **Gastric emptying** | 33±4 | 63±6  *** | 53±5  * | 55±3  * |
|  | **Intestinal emptying** | 90±6 | 105±4  * | 113±3  * | 110±7 |
|  | **Pellet Formation** | 93±3 | 105±4  * | 75±6  *** | 90±4  †††  ## |

**P*<0.05, ****P*<0.001, *****P*<0.0001, significantly different to DMSO-treated group.^†††^*P*<0.05, significantly different to 5-FU-treated group. ^##^*P*<0.05, ^####^*P*<0.0001, significantly different to BGP-15-treated group (n=5 mice/group).

**Supplementary Table S3 Speed of transit and emptying following 14 days repeated *in vivo* 5-FU+/-BGP-15 administration**

| **Parameters measured** | | **DMSO** | **5-FU** | **BGP-15** | **5-FU+BGP-15** |
| --- | --- | --- | --- | --- | --- |
| Speed of transit (time to reach each region, min) | **Stomach** | 0±0 | 0±0 | 0±0 | 0±0 |
|  | **Small Intestines** | 5±0 | 5±0 | 5±0 | 5±0 |
|  | **Caecum** | 77±5 | 83±3 | 60±5  **  †††† | 73±3  # |
|  | **Large Intestines** | 90±5 | 113±4  **** | 70±5  ***  †††† | 113±3  #### |
| Time for complete barium emptying (min) | **Gastric emptying** | 33±3 | 63±3  ** | 53±8  * | 60±6 |
|  | **Intestinal emptying** | 83±3 | 126±8  ** | 90±5  †††† | 140±0  ****  ### |
|  | **Pellet Formation** | 90±5 | 113±4  **** | 70±5  ***  †††† | 113±3  #### |

**P*<0.05, ***P*<0.01, ****P*<0.001, *****P*<0.0001 significantly different to DMSO-treated group. ^††††^*P*<0.0001, significantly different to 5-FU-treated group. ^#^*P*<0.05, ^###^*P*<0.001, ^####^*P*<0.0001, significantly different to BGP-15-treated group (n=5 mice/group).

**Supplementary** **Table S4 Faecal water content following 14 days repeated *in vivo* 5-FU+/-BGP-15 administration**

| **Parameters measured** | | **DMSO** | **5-FU** | **BGP-15** | **5-FU+BGP-15** |
| --- | --- | --- | --- | --- | --- |
| Wet weight (mg) | **Day 3** | 63.7±3.7 | 63.8±3.6 | 75.3±3.2  *  † | 68.0±3.4 |
|  | **Day 7** | 65.0±4.9 | 66.3±2.6 | 68.8±4.6 | 65.9±5.0 |
|  | **Day 14** | 63.7±6.7 | 76.9±4.5 | 67.9±4.3 | 51.7±5.1  ††  # |
| Dry Weight (mg) | **Day 3** | 28.1±1.4 | 32.6±1.3 | 30.2±1.3 | 26.3±1.3  † |
|  | **Day 7** | 30.5±1.7 | 33.1±1.5 | 32.9±2.8 | 27.6±1.7  † |
|  | **Day 14** | 33.0±4.2 | 37.2±2.1 | 32.7±1.8 | 19.7±1.5  †††  ### |
| Faecal water content (%) | **Day 3** | 56.0±1.0 | 52.8±2.3 | 59.6±1.5 | 59.9±3.7 |
|  | **Day 7** | 52.3±1.1 | 50.1±1.4 | 52.4±2.4 | 57.1±1.3  †† |
|  | **Day 14** | 49.3±1.7 | 54.8±1.4  ** | 54.2±2.4 | 60.7±1.7  † |

**P*<0.05, ***P*<0.01, significantly different to DMSO-treated group. ^†^*P*<0.05, ^††^*P*<0.01, ^†††^*P*<0.001, significantly different to 5-FU-treated group. ^#^*P*<0.05, ^###^*P*<0.001, significantly different to BGP-15-treated group (n=10 mice/group).

**Supplementary Table S5 Colonic motility following 14 days repeated *in vivo* 5-FU+/-BGP-15 administration**

| **Parameters measured** | | **DMSO** | **5-FU** | **BGP-15** | **5-FU+BGP-15** |
| --- | --- | --- | --- | --- | --- |
| Frequency (Number of Contractions/15 min) | **Total No# Contractions** | 28±1.6 | 34.9±3.3  ** | 25.7±2.6 | 72±4  ****  ††††  #### |
|  | **CMMCs** | 11.6±0.5 | 3.9±1.1  **** | 11.5±3.1 | 0.3±0  ****  †  # |
|  | **SCs** | 10.1±1.2 | 19.4±3.2  ** | 9.4±1.7  † | 39±5  **  †  ## |
|  | **FCs** | 6.3±0.8 | 11.4±1.3  ** | 4.9±1.7 | 33±5  **  †  # |
| Proportion | **CMMCs** | 43±3.1 | 14±4.1  ** | 47±10.7  †† | 0.4±0  ***  †  ### |
|  | **SCs** | 35±2.4 | 53±4.5  * | 36±5.8  † | 54±5  ****  # |
|  | **FCs** | 22±2.3 | 33±2.1  ** | 17±6.1  † | 46±5.4  *  # |

**P*<0.05, ***P*<0.01, ****P*<0.001, *****P*<0.0001, significantly different to DMSO-treated group. ^†^*P*<0.05, ^††^*P*<0.01, ^††††^*P*<0.0001, significantly different to 5-FU-treated group. ^#^*P*<0.05, ^##^*P*<0.01, ^###^*P*<0.001, ^####^*P*<0.0001, significantly different to BGP-15-treated (n=5 mice/group).
